# Disparate subcellular location of putative sortase substrates in *Clostridium difficile*

**DOI:** 10.1038/s41598-017-08322-1

**Published:** 2017-08-23

**Authors:** Johann Peltier, Helen A. Shaw, Brendan W. Wren, Neil F. Fairweather

**Affiliations:** 1 Centre for Molecular Bacteriology and Infection, Department of Life Sciences, Imperial College London, London, SW7 2AZ UK; 2 Department of Pathogen Molecular Biology, London School of Hygiene and Tropical Medicine, London, WC1E 7HT UK

## Abstract

*Clostridium difficile* is a gastrointestinal pathogen but how the bacterium colonises this niche is still little understood. Sortase enzymes covalently attach specific bacterial proteins to the peptidoglycan cell wall and are often involved in colonisation by pathogens. Here we show *C*. *difficile* proteins CD2537 and CD3392 are functional substrates of sortase SrtB. Through manipulation of the C-terminal regions of these proteins we show the SPKTG motif is essential for covalent attachment to the cell wall. Two additional putative substrates, CD0183 which contains an SPSTG motif, and CD2768 which contains an SPQTG motif, are not cleaved or anchored to the cell wall by sortase. Finally, using an *in vivo* asymmetric cleavage assay, we show that despite containing a conserved SPKTG motif, in the absence of SrtB these proteins are localised to disparate cellular compartments.

## Introduction

*Clostridium difficile* is an intestinal pathogen that causes significant mortality and morbidity throughout the world^1^. It is the leading cause of hospital acquired diarrhoea and has become increasingly common as a community acquired infection^2^. *C*. *difficile* produces two glycosylating toxins which play key roles in disease pathogenesis, targeting the gut epithelium resulting in severe inflammation and damage to the colon^1, 3^. Transmission of *C*. *difficile* is dependent on the production of highly resistant spores^4^ which germinate in the intestine. Normally the intestinal microbiota acts to suppress *C*. *difficile* proliferation but antibiotic treatment can modulate the host microbiota and can result in *C*. *difficile* growth, colonisation of the intestine and toxin production^5, 6^.

While details of the mechanisms and control of sporulation and toxin production are becoming increasingly understood, the processes involved in colonisation are still largely unclear. In many Gram-positive bacteria, surface structures such as pili, flagella and exposed surface proteins are implicated in colonisation and have been studied is some detail. The structural organisation of the *C*. *difficile* cell wall has been investigated^7^, and a diversity of factors that may impact on intestinal colonisation by *C*. *difficile* is now recognised^8^.

In many Gram-positive pathogens, the activity of sortases are often important in pathogenesis^9–12^. Sortases are enzymes which catalyse the cleavage and transpeptidation of specific protein substrates facilitating their covalent attachment to the peptidoglycan within the cell wall^13^. Cleavage and transpeptidation of sortase substrates occur at distinct motifs within a C-terminal cell wall sorting signal (CWSS)^14^ containing, in the case of sortase A from *Staphylococcus aureus*, an LPXTG motif. Variations of this motif are recognised by different classes of sortases and vary between species^15^. The CWSS comprises the LPXTG motif, followed by a hydrophobic region and a short, positively charged tail. Sortase substrates are targeted for the Sec secretion system by their N-terminal signal sequence, and are retained in the secretion machinery by the C-terminal hydrophobic domain and charged tail allowing sortase to cleave the LPXTG motif prior to transpeptidation^13^.

The genome of *C*. *difficile* strain 630 has a single sortase, SrtB, and several potential substrates with a variety of “LPxTG”-like motifs. SrtB has been shown to be functional in an *in vitro* cell free assay with short peptide substrates^16–18^ and to be required by *C*. *difficile* for the localisation of sortase substrates CD0386, CD2831 and CD3246^18, 19^. The motifs PPKTG, SPKTG, SPSTG and SPQTG present within *C*. *difficile* sortase substrates can be cleaved *in vitro*, while the NVQTG motif of the putative sortase substrate CD3145 is not cleaved, implying this protein is not a true sortase substrate^16, 18^. Moreover, structural insights into how *C*. *difficile* sortase specifically recognizes the peptide substrate PPKTG have recently been provided^20^. The protease PPEP-1 (CD2830/Zmp1) has been shown to further cleave substrates CD2831 and CD3246 at a position N-terminal to the CWSS, releasing these proteins into the culture supernatant^19, 21, 22^. Cleavage by PPEP-1 has been shown to be tightly regulated by c-di-GMP which regulates both PPEP-1 and CD2831 expression^19^, with a c-di-GMP controlled self-splicing ribozyme controlling CD3246 expression^23^. Additionally, small RNAs have been shown to control expression of the putative sortase substrate CD0183^24^. The sortase substrate CD0386 is found on the conjugative transposon CTn1 in *C*. *difficile* 630, while its homologue CD3392 is found on CTn7, with similar genes found in transposons of other strains of *C*. *difficile* ^25^. A PPEP-1mutant exhibits increased collagen binding activity, implicating sortase substrates CD2831 and CD3246 in this phenotype^22^. Additionally, CD3246 is predicted to form an internal thioester bond which may be involved in binding to host proteins^26^.

In this study we confirm the *in vivo* cellular activity in *C*. *difficile* of sortase SrtB in strain 630 on substrates containing the motif SPKTG, while those containing SPSTG and SPQTG motifs are uncleaved and we characterise disparate cellular localisation phenotypes of sortase substrates.

## Results

### CD3392 is cell wall protein anchored by sortase

**Figure 1:**
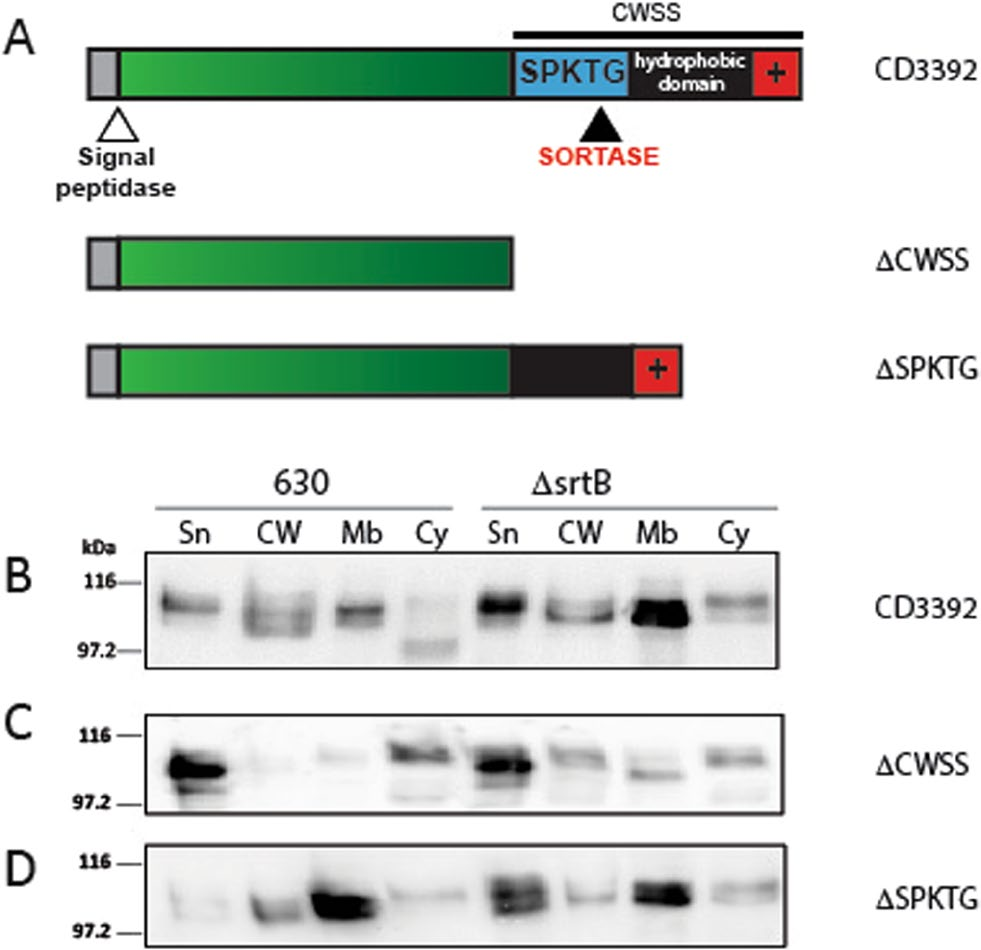
The SPKTG motif is essential for processing of CD3392 by sortase. (**A**) Schematic representation of putative sortase substrate CD3392 and deletion derivatives lacking the entire cell wall sorting signal (ΔCWSS) or the SPKTG sorting motif (ΔSPKTG). The signal peptide (grey), mature protein (green), C-terminal sorting signal comprising the SPKTG motif, hydrophobic domain (black) and charged tail (+) are shown. Cleavage sites for signal peptidase (white arrow) and sortase (black arrow) are shown. (**B**–**D**) Cells of wild type 630 and the Δ*srtB* mutant carrying pJKP036 expressing full length CD3392 (**B**), and derivatives of CD3392 lacking the cell wall sorting signal (pJKP051; ΔCWSS) (**C**) or the SPKTG sorting motif (pJKP046; ΔSPKTG) (**D**) were fractionated into supernatant (Sn), cell wall (CW), membrane (Mb) and cytoplasm (Cy) compartments. Protein fractions were separated by SDS-PAGE and visualised by western blot using anti-CD3392 antibody. Western blots were cropped for clarity; uncropped images can be found in Supplementary Fig. S1.

**Figure 2:**
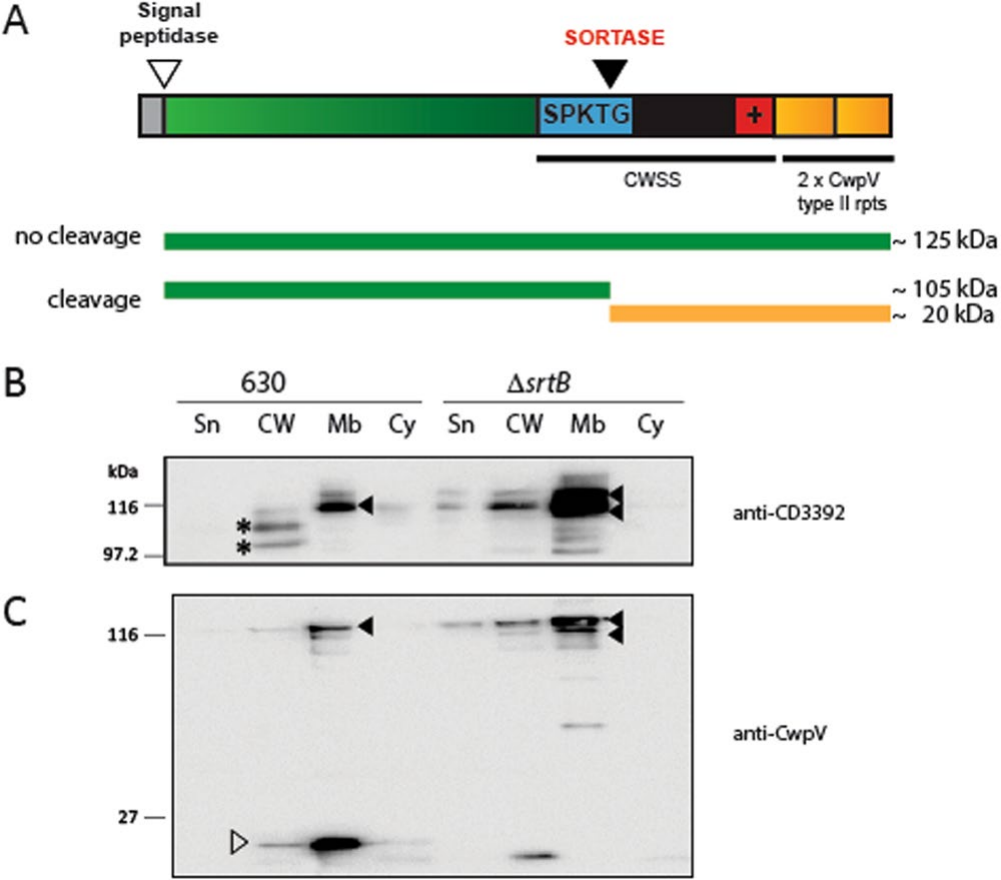
Sortase cleaves fusion protein CD3392-CwpV for cell wall attachment. (**A**) Schematic representation of CD3392-CwpV fusion protein and the predicted cleavage products. Structural domains of CD3392 are as in Fig. 1, with additional Type II CwpV repeats at the C-terminus (yellow). The ~125 kDa green bar represents uncleaved CD3392-CwpV fusion protein, while a green (~105 kDa) and yellow (~20 kDa) bars indicate predicted cleavage products following sortase activity. (**B**,**C**) Cells of wild type 630 and the Δ*srtB* mutant carrying pJKP048 and expressing the CD3392-CwpV fusion protein were fractionated into supernatant (Sn), cell wall (CW), membrane (Mb) and cytoplasm (Cy). Protein fractions were analysed by western blot using antibody against CD3392 (**B**) or against CwpV Type II repeats (**C**). ◀, uncleaved CD3392-CwpV; *, large CD3392-CwpV cleavage product; ▷, 20 kDa fragment containing CwpV repeats. Western blots were cropped for clarity; uncropped images can be found in Supplementary Fig. S2.

CD3392, a putative collagen binding protein, displays a high sequence identity of 94.38% to the known *C*. *difficile* sortase substrate CD0386^18^. These proteins contain the same sorting motif, SPKTG, but otherwise the C-terminal sequences are distinct and could affect the sorting of this protein (Table 1). CD3392 has the classic structure of sortase substrates with an N-terminal secretion signal, and a C-terminal cell wall sorting signal (CWSS) comprising an “LPXTG” like motif, a hydrophobic region and a charged tail (Fig. 1A). Under laboratory conditions, CD3392 is not expressed to a level detectable by antibody against recombinant CD3392. The *CD3392* gene was therefore expressed on plasmid pJKP036 under control of an anhydrotetracycline inducible promoter in *C*. *difficile* 630 and in a *srtB* knockout strain, Δ*srtB* ^19^. The distribution of CD3392 within cytosolic, membrane and cell wall (peptidoglycan) fractions in the two strains induced with 100 ng/ml of ATc was studied. The cell wall fraction was generated using the purified catalytic domain of endolysin CD27L^27^ in sucrose buffer to prevent cell lysis of the protoplasts. This technique efficiently and reliably hydrolyses the cell wall peptidoglycan and therefore ensures complete separation of the cell wall and membrane fractions^19^. CD3392 is found in all cell compartments in the wild type 630 strain (Fig. 1B). In the Δ*srtB* mutant, there is an increased level of CD3392 in the supernatant and a large accumulation of CD3392 in the membrane, demonstrating that its localisation is dependent on the activity of sortase. In the absence of sortase, the CWSS is not cleaved and its hydrophobic domain can therefore retain CD3392 within the membrane. Some protein that is not retained and passes through the membrane and is secreted into the culture supernatant. Secretion implies that proteins also need to cross the cell wall, and bands still observed in the cell wall fraction of the Δ*srtB* mutant likely correspond to residual un-cleaved CD3392 retained in the cell wall in a non-covalent manner (see Fig. 2 below). To determine whether the CWSS motif is involved in the interaction with sortase, a series of C-terminal deletions were expressed in wild type 630 and Δ*srtB* (Fig. 1A). When the entire CWSS is deleted (ΔCWSS), CD3392 is found predominantly in the supernatant in both strains (Fig. 1C) showing the CWSS is necessary for cell association. When only the SPKTG motif is deleted (ΔSPKTG), and the hydrophobic domain and charged tail are retained, CD3392 is found trapped in the cell membrane fraction in both strains (Fig. 1D), mimicking the phenotype of the *srtB* knockout. These data confirm that the SPKTG motif is necessary for processing of CD3392 by sortase and that the hydrophobic region and charged tail are required for membrane association to enable sorting.

**Table 1:** C-terminus analysis of putative sortase substrates in *C*. *difficile*.

Gene^a^	“LPxTG”^b^	Hydrophobic domain^c^	Charged tail^d^
CD0183	**…SPSTG**		
CD2537	**…SPKTG**		
CD2768	**…SPQTG**		
CD2831	**…PPKTG**		
CD3145	**…NVQTG**		
CD3246	**…SPKTG**		
CD0386	**…SPKTG**		
CD3392	**…SPKTG**		
SPA	**…LPETG**		

^a^Genes and corresponding proteins are from strain 630 (top) and from *S*. *aureus* Protein A (SPA); ^b–d^the conserved LPXTG motif (bold), the hydrophobic domain (underlined) and the positively charged tail (italics, bold) are indicated.

### The SPKTG motif of CD3392 is cleaved by sortase

As the SPKTG motif was found to be necessary for cell wall association, a novel asymmetric cleavage assay was designed to determine whether sortase catalyses cleavage of this motif *in vivo*. CwpV is a *C*. *difficile* surface protein which contains repeating units, falling into five known classes, or types, which are antigenically distinct^28^. Strain 630 expresses Type I CwpV repeats, therefore the antigenically distinct Type II CwpV repeats from strain R20352 were utilised for a cleavage assay. CD3392 was cloned with the addition of two Type II repeats at the C-terminus (Fig. 2A). The full length CD3392-CwpV fusion protein is predicted to migrate at ~125 kDa, with the cleavage fragments representing the majority of CD3392 at ~105 kDa, and the CD3392 C-terminal region plus CwpV repeats at ~20 kDa. The CD3392-CwpV fusion protein was expressed in wild type 630 and in the Δ*srtB* mutant. As expected, CD3392-CwpV is found substantially accumulated in the membrane compartment of the *srtB* mutant, as shown by immunoblotting with anti-CD3392 antibodies (Fig. 2B, black arrows), reinforcing the requirement of SrtB for translocation of CD3392 through the membrane. Low levels of CD3392-CwpV are observed in the membrane fraction of the wild-type strain (Fig. 2B, black arrows), probably because wild-type levels of SrtB are not able to cope with the artificially high levels of CD3392-CwpV. Faint lower molecular weight bands, that could correspond to cleaved CD3392, are also observed in the cell wall of wild type cells (asterisks) but are absent in the *srtB* mutant. To more clearly distinguish between the cleaved and uncleaved forms of CD3392-CwpV, the C-terminal CwpV repeats were detected using a specific antibody. As shown in Fig. 2C, cleaved CwpV repeats migrating at ~20 kDa can be observed predominantly in the membrane fraction of wild type cells. This supports the model whereby the C-terminal hydrophobic domain and charged tail act as a membrane anchor to enable sorting. In contrast, cleaved CwpV repeats are not detected in any subcellular fraction of the *srtB* mutant. A minor low molecular weight band can be seen in the cell wall fraction of the *srtB* mutant, but it is smaller than the species observed in wild type cells and is likely the result of proteolysis. Meanwhile, the high molecular weight band representing uncleaved CD3392-CwpV is observed in the membrane of the *srtB* mutant at high levels and in the membrane of the wild type cells at lower levels. Additionally a full length CD3392-CwpV band is observed in the cell wall of Δ*srtB*, confirming the speculation in Fig. 1B that the species observed in the cell wall of this mutant was unprocessed CD3392. These results clearly confirm that sortase cleaves CD3392 for processing from the membrane onto the cell wall.

### Covalent attachment of CD2537 to the cell wall is dependent on its SPKTG motif and sortase processing

**Figure 3:**
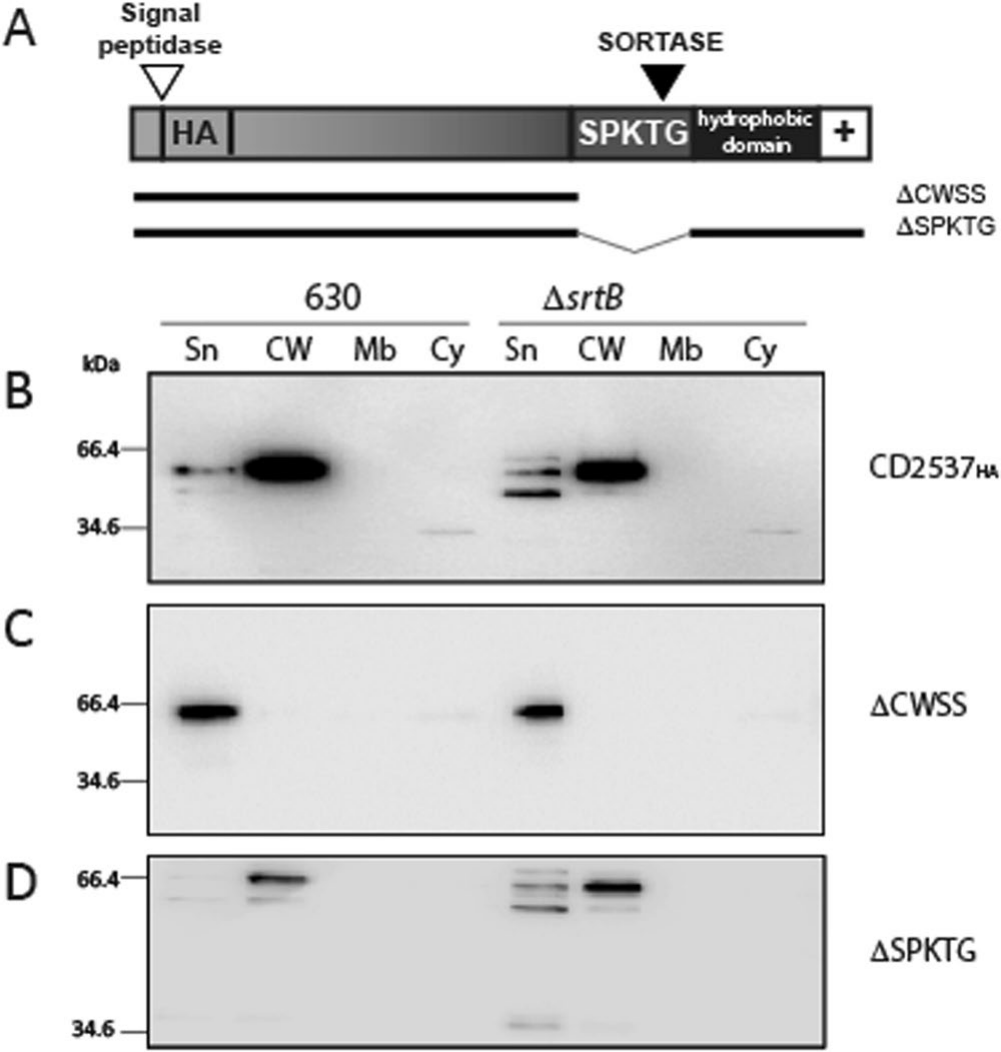
CD2537 cell wall association can occur independently of sortase activity. (**A**) Schematic of putative sortase substrate CD2537_HA_ protein and predicted cleavage sites with derivatives indicated by bars. Wild type 630 and the Δ*srtB* mutant carrying pJKP064 expressing full length CD2537_HA_ (**B**), and derivatives lacking the cell wall sorting signal (pJKP071; ΔCWSS) (**C**), or the SPKTG sorting motif (pJKP072; ΔSPKTG) (**D**) were fractionated into supernatant (Sn), cell wall (CW), membrane (Mb) and cytoplasm (Cy) compartments. Proteins were visualised by western blot with anti-HA tag antibody. Western blots were cropped for clarity; uncropped images can be found in Supplementary Fig. S3.

CD2537 is the final putative sortase substrate in *C*. *difficile* 630 containing an SPKTG motif with the Phobius webserver predicting a weak signal peptide from residues 1 to 20^29^. An HA tag was added at the N-terminus of CD2537 between amino acids 26 and 27, and the gene was cloned under a tetracycline inducible promoter and introduced into wild type 630 (Fig. 3A). CD2537 was predominantly found in the cell wall, strongly suggesting the presence of a functional signal peptide. A similar subcellular location of CD2537 was observed in Δ*srtB* with no accumulation in the membrane (Fig. 3B). When the entire CWSS motif was deleted, CD2537 was found in the supernatant in both strains (Fig. 3C), suggesting that this domain is necessary for cell wall association. When only the SPKTG motif is deleted, CD2537 is again found associated with the cell wall in both wild-type and mutant strains (Fig. 3D), suggesting that the hydrophobic domain and charged tail are involved in association with the cell wall independently of sortase activity.

**Figure 4:**
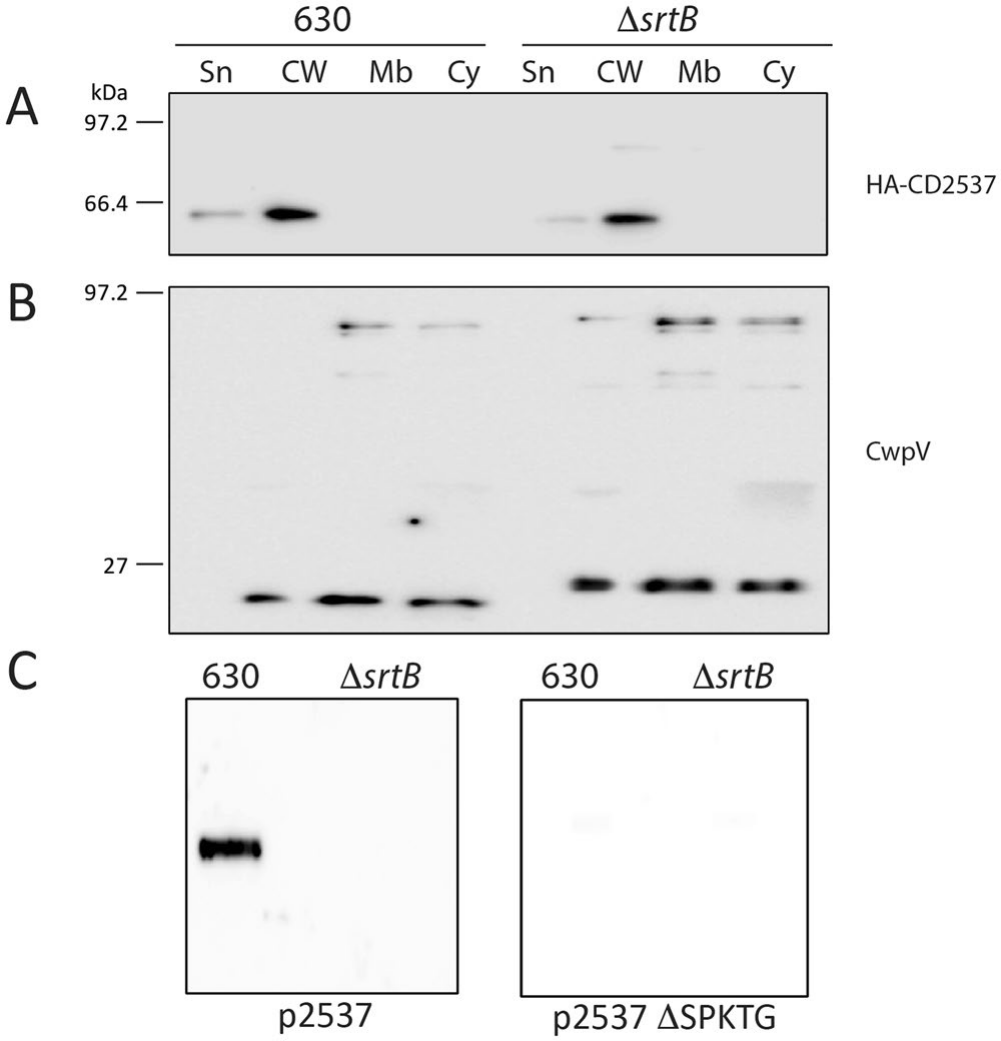
Sortase covalently anchors CD2537 to the peptidoglycan. Cells of wild type 630 and the Δ*srtB* mutant expressing plasmid-encoded CD2537_HA_-CwpV fusion protein were fractionated into supernatant (Sp), cell wall (CW), membrane (Mb) and cytoplasm (Cy) compartments. Protein fractions were analysed by western blot using anti-HA antibody (**A**) or anti-CwpV Type II repeats antibody (**B**). Peptidoglycan fractions were purified from wild type 630 and the Δ*srtB* mutant expressing full length CD2537_HA_ or its ΔSPKTG derivative and analysed by dot blot with anti-HA tag antibodies to detect the presence of CD2537 covalently anchored to peptidoglycan (**C**). Western blots were cropped for clarity; uncropped images can be found in Supplementary Fig. S4.

To determine whether the protein localised to the cell wall of Δ*srtB* was full length CD2537 or whether further processing occurs, CD2537 was expressed as a fusion protein with CwpV repeats at the C-terminus. A cleavage event is observed in both wild type 630 and Δ*srtB*, with cleaved CD2537 localised to the cell wall fraction (Fig. 4A). However, the cleaved CwpV fragment migrates at a larger size in Δ*srtB* compared with wild type cells (Fig. 4B), which suggests that an alternative cleavage event can occur in the absence of sortase to release CD2537 from the membrane. The size of this fragment suggests that a site upstream of the SPKTG motif may be recognised by another enzyme to release CD2537 from the membrane in the absence of sortase.

The cell wall extraction method utilised releases all proteins external to the cytoplasmic membrane, including non-covalently anchored proteins such as the S-layer proteins. To assess whether CD2537 is covalently associated with the cell wall fraction, peptidoglycan was purified from wild type 630 and the *srtB* mutant with only covalently linked molecules retained, and a dot blot undertaken to detect CD2537. Figure 4C shows that in wild type cells CD2537 is found in the pure peptidoglycan fraction, whereas it is absent from this fraction in Δ*srtB*. When the SPKTG motif is deleted from CD2537, the protein is not found covalently attached to the peptidoglycan in either strain. This shows that CD2537 is covalently anchored to the cell wall by sortase through its SPKTG motif, but in the absence of sortase it can associate with the cell wall in a non-covalent manner by an alternative processing reaction.

### CD0183 and CD2768 are not processed by sortase in *C. difficile*

**Figure 5:**
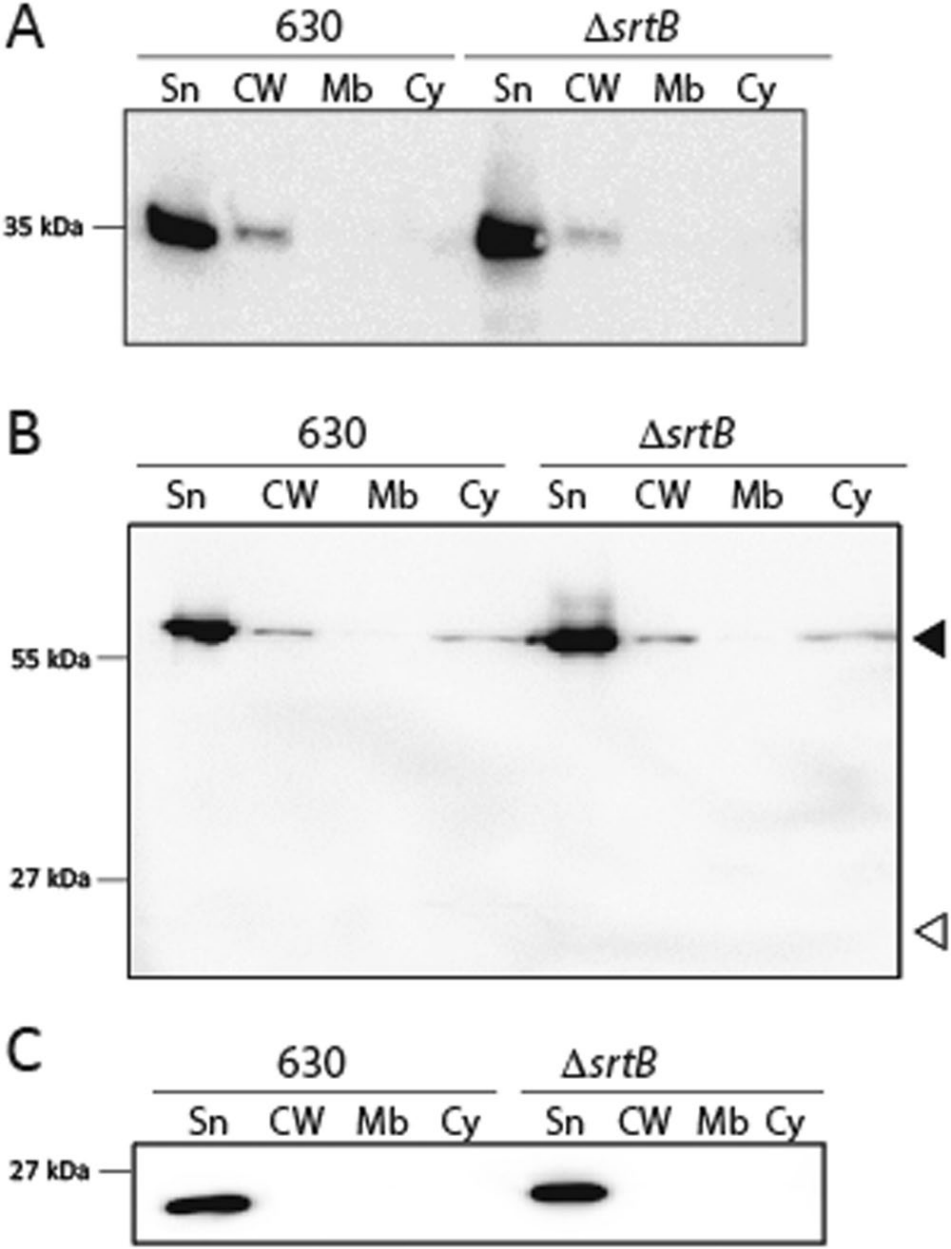
CD0183 and CD2768 are not anchored to the peptidoglycan by sortase in *C*. *difficile*. Cells of wild type 630 and the Δ*srtB* mutant without plasmid (**A**), expressing plasmid encoded CD0183-CwpV fusion protein (pHAS039) (**B**), or plasmid encoded CD2768_HA_ (pJKP077) (**C**) were fractionated into supernatant (Sn), cell wall (CW), membrane (Mb) and cytoplasm (Cy) compartments. Protein fractions were analysed by western blot using anti-0183 antibody (**A**), anti-CwpV antibody (**B**), or anti-HA tag antibody (**C**). ◀, uncleaved CD0183-CwpV; ◁, size of expected fragment containing CwpV repeats. Western blots were cropped for clarity; uncropped images can be found in Supplementary Fig. S5.

Previous studies have shown that, *in vitro*, SPKTG and SPSTG motifs are cleaved by sortase with comparable efficiency^16^. To determine whether the SPSTG motif is utilised by sortase *in vivo*, we investigated the localisation of CD0183 in *C*. *difficile*. Expression levels of CD0183 in *C*. *difficile* 630 are sufficient to detect efficiently by immunoblotting. In both wild type 630 and Δ*srtB*, CD0183 is observed principally in the culture supernatant with a minor band detectable in the cell wall (Fig. 5A), suggesting either that CD0183 is not processed by sortase, or it is further processed from the cell wall as seen with CD2831 and CD3246^19^. To determine whether further processing was occurring, CD0183 was cloned on a plasmid with two type II CwpV repeats at its C-terminus and cleavage of this fusion protein was analysed. As shown in Fig. 5B, uncleaved CD0183-CwpV is detected in the culture supernatant and cell wall in both wild type 630 and Δ*srtB*, with no evidence of cleavage products. This shows that despite the ability of sortase to cleave the SPSTG motif *in vitro*, this motif in CD0183 is not processed by sortase in *C*. *difficile* cells.

The last putative sortase substrate, CD2768, harbours an SPQTG motif, which has been shown to be cleaved *in vitro* with greater efficiency than the SPKTG motif^16^. Since no antibody against CD2768 was available, a derivative of *CD2768* with an HA-tag inserted immediately C terminal of its signal peptide was constructed on a plasmid under control of the anhydrotetracycline promoter. Upon induction, HA-CD2768 is detected by Western blot and exclusively observed in the culture supernatant of both wild type and Δ*srtB* strains (Fig. 5C). This result strongly suggests that, as with CD0183, this protein is not processed by sortase.

## Discussion

The genome of *C*. *difficile* strain 630 contains a single functional sortase enzyme (SrtB) and eight potential sortase substrates containing a variety of sorting motifs^16^; (Table 1). Of these substrates, only CD0386, CD2831 and CD3246 have been shown to be processed by SrtB *in vivo* ^18, 19^ and the collagen binding protein CD3145 (CbpA) is not processed by sortase *in vitro*, suggesting it is not a true substrate *in vivo* ^16, 18, 30^. Additionally, surface localisation of CD2831 and CD3246 is further modulated by the activity of the proteolytic activity of PPEP-1 that can release attached proteins from the cell wall in a process regulated by cyclic-di GMP^19, 21, 22^. Thus, expression of sortase and the presence of a CWSS in a surface protein is clearly not sufficient to localise substrates to the cell wall in *C*. *difficile*. This situation prompted us to investigate further sortase substrates to characterise their cellular localisation.

We chose to study the substrates CD3392 and CD2537, both containing an SPKTG sorting motif. With the aid of protein modifications, we show that CD3392 displays a classical “sorted” phenotype; that of being cell wall attached in the presence of sortase but accumulated in the membrane in the absence of a functional sortase enzyme. However, it was somewhat surprising that the total levels of CD3392 observed in the wild type strain were much lower than those found in the sortase mutant. In the wild type strain, we would have expected increased amounts of CD3392 to be present at the cell surface, but we did not observe this. This may suggest that extensive degradation of CD3392 by unknown proteases may occur once it localised at the cell surface.

**Figure 6:**
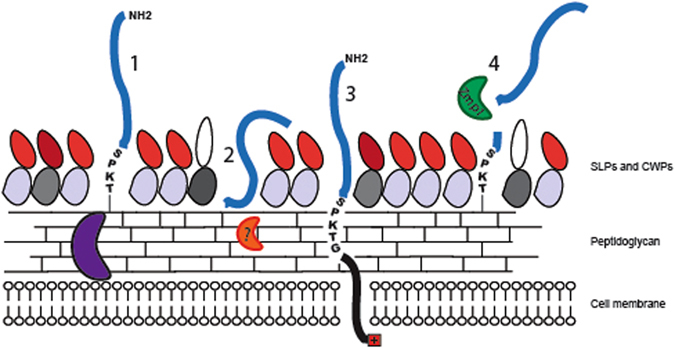
SPKTG sortase substrate processing in *C*. *difficile*. A functional model of sortase substrate processing to the cell wall of *C*. *difficile*. The surface of *C*. *difficile* is comprised of a thick peptidoglycan cell wall (bars), secondary cell wall polymers (not shown) and an external, non-covalently attached S-layer comprised of S-layer protein SlpA (grey and red) and an assortment of minor cell wall proteins (CWPs). Sortase (purple) is shown at the membrane adjacent to a peptidoglycan anchored sortase substrate (1). Through degradation or release by unknown proteases (orange) or non-specific proteolysis, some un-sorted substrates as seen with CD2537 in the absence of sortase are released from the membrane and are non-covalently associated with the cell wall (2). CD3392 remains predominantly anchored in the membrane if sortase is not available to covalently attach it to the cell wall (3). Proteases such as PPEP-1 (green) are able to cleave sortase substrates CD2831 and CD3246 from the cell wall for release into the supernatant (4).

CD2537 was found covalently anchored to the peptidoglycan through the activity of SrtB, yet we also found CD2537 still able to associate with the cell wall in the absence of sortase, however, this association was shown to be non-covalent. Some sortase substrates have previously been shown to associate at low levels with cells in sortase mutants, for example the GamR protein of *Bacillus anthracis* associates with cells during peptidoglycan purification until the final stages when it is absent from the pure peptidoglycan fraction^31^. It is interesting to note that this processing towards a non-covalent interaction does not seem to occur in wild-type *C*. *difficile* 630 expressing sortase. This may be due to protection from proteomic “shaving” as observed with the *B*. *subtilis* sortase substrate YfkN which is only significantly released from the cells in the absence of sortase^32^ or may just be a result of protein degradation from the secretion machinery. It remains to be determined which, if any, enzyme is capable of processing membrane bound CD2537. Our results show that the localisation phenotype in *C*. *difficile* in the absence of sortase varies for each sortase substrate, even within the subset of SPKTG motif proteins (Fig. 6).

Despite *C*. *difficile* SrtB being able to cleave *in vitro* synthetic peptides bearing SPSTG and SPQTG motifs, CD0183 and CD2768, which harbour these two motifs respectively, do not appear to be cell wall anchored. Moreover, full length CD0183 protein fused to CwpV repeats was not processed in *C*. *difficile* cells. A common feature between CD0183 and CD2768 is that they contain a weakly hydrophobic C-terminal domain and have a short positively charged tail (Table 1). Since the function of these two elements is to delay secretion across the membrane and facilitate processing by sortase^13^, it is possible that the proteins are not retained long enough for sortase processing. In *Staphylococcus aureus*, protein A (Spa) is predominantly anchored to the cell surface by sortase A but a significant proportion of this protein is also released in the culture supernatant^33^. It was recently shown that, after its covalent anchoring to peptidoglycan, Spa protein together with linked peptidoglycan fragments can be shed from the cell surface into the extracellular medium by the peptidoglycan hydrolase LytM. Therefore, Spa released by this mechanism is firstly processed by SrtA to be covalently anchored to peptidoglycan and subsequently released into the supernatant. In addition, full length Spa protein with an unprocessed sorting signal has also been observed in the culture supernatant^34^. Replacing the Spa sorting signal with the sorting signal of another SrtA substrate, SrdE, reduces the amount of Spa released, demonstrating that release of a sortase substrate depends on the sequence of its sorting signal.

The data presented here shed light on the nature of proteins recognized by sortase in *C*. *difficile*. Despite our detailed knowledge of the mechanism of sortase cleavage, our knowledge of the role of sortase in *C*. *difficile* is in its infancy, particularly with regards to pathogenesis as published studies have only reported on the hamster model of pathogenic, toxigenic infection^18^. Sortase is known to act on protein CD2831 to mediate surface localisation, which may facilitate adhesion to substrates in the intestinal tract^21, 22^. This surface attachment is regulated by the protease activity of PEEP-1, which is under the control of cyclic di-GMP. Further knowledge of the activities of the sortase substrates combined with *in vivo* experiments using relevant animal models such as colonisation will throw light on the role(s) of sortase and its substrates during infection.

In conclusion, it is clear that possession of a sortase CWSS does not necessarily result in permanent covalent attachment of proteins to the Gram-positive cell wall, even when cleavage activity is observed *in vitro*. Furthermore, as shown by several studies^19, 33, 34^, release of sorted proteins from the cell wall appears to be a strategy used by several species to present proteins either on the cell surface or in the supernatant.

## Experimental Procedures

### Strains, plasmids and growth conditions

*C*. *difficile* 630^35^ and a previously constructed Δ*srtB* mutant^19^ were grown under anaerobic conditions (80% N_2_, 10% CO_2_, 10% H_2_) in an anaerobic incubator (Don Whitley Scientific) at 37 °C on Brain Heart Infusion Supplemented (BHIS) plates or in broth [BHI supplemented with 5 g/l yeast extract (BD Bacto), and 1 g/l L-cysteine, and 15 g/l agar (Bacto) for plates]. Growth was supplemented with thiamphenicol (15 μg/ml) for plasmid propagation, cycloserine (250 μg/ml) and cefoxitin (25 μg/ml) for counter selection against *E*. *coli* during conjugation, and anhydrotetracycline (ATc 100 ng/ml) for induction of *P*_*tet*_ expression vectors^36^. *Escherichia coli* NEB5α (New England Biolabs) was used for cloning and plasmid propagation, strain CA434 (HB101 carrying R702) was used for conjugation of plasmids into *C*. *difficile*, and Rosetta (Novagen, carrying pRARE) was used for expression of proteins for purification. *E*. *coli* strains were grown on LB agar and in LB broth supplemented with chloramphenicol (15 μg/ml) and kanamycin (50 μg/ml).

### Construction of plasmids

Plasmids and oligonucleotides are listed in Table S1 and were constructed using standard methods. For expression of recombinant CD0183 (residues 25–291) and CD3392 (residues 30–294), primers NF1612 with NF1613 and NF1616 with NF1617 respectively were used to PCR-amplify the corresponding DNA sequence. Purified PCR products were digested with NcoI and XhoI and ligated into the pET28a vector digested with the same enzymes, yielding pHAS026 and pHAS028. These pET28a plasmids were initially transformed into *E*. *coli* strain NEB5α and sequences verified prior to transformation into *E*. *coli* strain Rosetta for protein expression and purification.

To introduce CwpV repeats at the C-terminus of CD0183, primers NF1785 and NF1817 were paired to amplify the entire coding sequence of CD0183 and its ribosome binding site without its gene stop codon from 630 genomic DNA and include an XhoI site for insertion of CwpV repeats between XhoI and BamHI sites. Purified PCR product was digested with SacI and BamHI and ligated into pRPF185 to create pHAS037. CwpV was PCR-amplified from R20352 genomic DNA by primers NF1818 and NF1799, and the purified PCR product was digested with XhoI and BamHI for ligation into pHAS037, yielding pHAS039.

The entire coding sequence of CD2537 and its ribosome binding site were amplified from 630 genomic DNA with primers NF2643 and NF2288, digested with SacI and BamHI and ligated into pRPF185 to create pJKP053. To introduce an hemagluttinin epitope tag (HA-tag) into CD2537, plasmid pJKP053 was then modified by inverse PCR with oligonucleotides NF2722 and NF2723, containing an appended sequence encoding for the HA tag, yielding pJKP064. The HA-tag was introduced downstream of the signal peptide. To remove the SPKTG motif from *CD2537*, primers NF2855 and NF2856 were paired for inverse PCR from pJKP064 to create pJKP071. To remove the entire cell wall sorting motif from CD2537, primers NF2855 and NF2857 were paired for inverse PCR from pJKP064 to create pJKP072. To introduce CwpV repeats at the C-terminus of CD2537, primers NF2643 and NF2863 were paired to amplify the entire coding sequence of HA-CD2537 and its ribosome binding site without its gene stop codon from pJKP064. Purified PCR product was digested with SacI and XhoI and ligated into pHAS039 backbone digested with the same enzymes to create pJKP074.

The entire coding sequence of CD2768 and its ribosome binding site were amplified from 630 genomic DNA with primers NF2883 and NF2884, digested with SacI and BamHI and ligated into pRPF185 to create pJKP075. To introduce an hemagluttinin epitope tag (HA-tag) into CD2768, plasmid pJKP075 was then modified by inverse PCR with oligonucleotides NF2887 and NF2888, containing an appended sequence encoding for the HA tag, yielding pJKP077. The HA-tag was introduced downstream of the signal peptide.

The entire coding sequence of CD3392 and its ribosome binding site were amplified from 630 genomic DNA with primers NF2378 and NF2379, digested with SacI and BamHI and ligated into pRPF185 to create pJKP036. To remove the SPKTG motif from CD3392, primers NF1648 and NF2582 were paired for inverse PCR from pJKP036 to create pJKP046. To remove the entire cell wall sorting motif from CD3392, primers NF2581 and NF2582 were paired for inverse PCR from pJKP036 to create pJKP051. To introduce CwpV repeats at the C-terminus of CD3392, primers NF2134 and NF2135 were paired to amplify the entire coding sequence of CD3392 and its ribosome binding site without its gene stop codon from pJKP036. Purified PCR product was digested with SacI and XhoI and ligated into pHAS039 backbone digested with the same enzymes to create pJKP048.

### Purification of recombinant CD0183 and CD3392 protein and generation of antibodies

Protein expression was induced in *E*. *coli* Rosetta using OverNight Express Instant TB medium (Merck). Recombinant proteins were purified by affinity chromatography in a HisTrap chelating HP column (GE Healthcare) on an AKTA liquid chromatography system (Pharmacia). Purified CD0183 and CD3392 protein were injected into BALB/c mice together with Freund’s incomplete adjuvant to raise polyclonal antibodies.

### Preparation and analysis of cell extracts

Whole cell lysates of *C*. *difficile* were prepared as previously described^36^. Briefly, cultures were harvested by centrifugation at 4,000 × *g* for 10 min at 4 °C and the pellets were frozen at −20 °C. Cells were thawed, resuspended in PBS containing 40 μg/ml DNase I to an *A*_600 nm_ of 20, and incubated at 37 °C for 40 min. Culture supernatants were filtered with a 0.22-*μ*m filter and precipitated on ice with 10% TCA for 30 min. Precipitates were harvested at 25,000 × *g* for 10 min at 4 °C, and the pellet was washed twice in ice-cold 90% acetone for 10 min. The pellet was finally resuspended in PBS to an *A*_600 nm_ of 20. Cell wall proteins were extracted as previously described^19^. Briefly, *C*. *difficile* cultures were harvested by centrifugation at 4,000 × *g* for 2 min, resuspended in phosphate-sucrose buffer (0.05 M HNa_2_PO_4_, pH 7.0, 0.5 M sucrose) to an *A*_600 nm_ of 20 with 30 μg/ml purified CD27L endolysin^19^, and incubated at 37 °C for 1 h. Protoplasts were recovered by centrifugation at 6000 × *g* for 20 min at 4 °C. Supernatants containing the cell wall (CW) fraction were removed, and the protoplast pellet was lysed with resuspension in phosphate buffer (0.05 M HNa_2_PO_4_, pH 7.0) containing 40 μg/ml DNase I at an *A*_600 nm_ of 50 and incubated at 37 °C for 45 min. Lysates were harvested at 25,000 × *g* for 10 min at 4 °C, supernatants containing the cytoplasmic (Cy) fraction were removed, and the membrane pellet was resuspended in phosphate buffer with 1% SDS at an *A*_600 nm_ of 20. For analysis by SDS-PAGE, an equal volume of 2 × SDS sample buffer was added to protein samples. SDS-PAGE and Western immunoblotting were carried out using standard methods.

### Peptidoglycan purification

Cultures of *C*. *difficile* were harvested by centrifugation at 5000 × *g* for 10 min at 4 °C and the pellets were resuspended in PBS to an *A*_600 nm_ of 40. Cells were lyzed through 3 passages in French press and cell lysates were centrifuged at 25000 × *g* for 20 min at 4 °C. The pellets were then resuspended in PBS containing 1% SDS to an *A*_600 nm_ of 40 and the suspensions were boiled for 15 min. After centrifugation at 25000 × *g* for 20 min at room temperature, the pellets were resuspended again in PBS containing 1% SDS to an *A*_600 nm_ of 40 and boiled for 10 min. After centrifugation at 25000 × *g* for 20 min at room temperature, the pellet was washed twice with PBS and finally resuspended in PBS to an *A*_600 nm_ of 40. The suspension contains purified peptidoglycan and its covalently-attached molecules. Peptidoglycan fractions were analysed by dot-blot using a Bio-Dot apparatus (Biorad).

## Electronic supplementary material

Supplementary information

2 supplementary pdf
