# Supplementary material for: Disparate subcellular location of putative sortase substrates in Clostridium difficile
Source: Sci Rep. 2017 Aug 23;7:9204. doi: 10.1038/s41598-017-08322-1 (PMC5569036; doi:10.1038/s41598-017-08322-1)
Supplement: Supplementary file 1 — Supplementary information [file 41598_2017_8322_MOESM1_ESM.pdf]

Table S1. Plasmids and oligonucleotide primers used.

**Plasmid or primer**	**Characteristic/description**	**Source/construction**
**Plasmids**		
pET28a	*E. coli* expression vector. Kan^R	Novagen
pRPF185	*E. coli*-*C. difficile* shuttle vector for protein expression. P_{tet}-*gusA*, Tm^R	(1)
pHAS026	CD0183 (25-291)-His$_6$ in pET28a	This study
pHAS028	CD3392 (30-294)-His$_6$ in pET28a	This study
pHAS035	pRPF185 derivative carrying P_{tet}-*CD0183*	This study
pHAS037	pHAS036 with stop codon removed and XhoI site introduced in front of BamHI	This study
pHAS039	pHAS037 with CwpV Type II x 2 repeats fused at the C-term extremity	This study
pJKP036	pRPF185 derivative carrying P_{tet}-*CD3392*	This study; Fig 1
pJKP046	pJKP036 with SPKTG motif of CD3392 removed	This study Fig 1
pJKP048	pRPF185 derivative carrying P_{tet}-*CD3392* with CwpV Type II x 2 repeats fused at the C-term extremity	This study
pJKP051	pJKP036 with entire sorting signal of CD3392 removed	This study Fig 1
pJKP053	pRPF185 derivative carrying P_{tet}-*CD2537*	This study
pJKP064	pJKP053 with addition of a HA-tag encoding sequence into *CD2537*	This study Fig 4
pJKP071	pJKP064 with SPKTG motif of CD2537 removed	This study Fig 4
pJKP072	pJKP064 with entire sorting signal of CD2537 removed	This study Fig 4
pJKP074	pRPF185 derivative carrying P_{tet}-*HA-CD2537* with CwpV Type II x 2 repeats fused at the C-term extremity	This study
pJKP075	pRPF185 derivative carrying P_{tet}-*CD2768*	This study
pJKP077	pJKP075 with addition of a HA-tag encoding sequence into *CD2768*	This study
**Primer**	**Sequence (5' to 3')***	**Characteristics or use**
NF1612	GGGCCATGGATGATGAAGTAAATGATTCATCTCAAAATAAGG	Amplify *CD0183* for pET28a cloning

NF1613	GGGCTCGAGAGTTGTGAAGAATACTAAGTCTCCTG	Amplify *CD0183* for pET28a cloning
NF1616	GGGCCATGGATGAAAGTAAGCAATACTGGACGGAAAG	Amplify *CD3392* for pET28a cloning
NF1617	GGGCTCGAGATCTGATACTTTCAAGGTCGCTGTTTC	Amplify *CD3392* for pET28a cloning
NF1648	GACAGTACAAACCTTATGGCTTTTAT	Remove SPKTG sorting motif of *CD3392* by inverse PCR
NF1785	GAGCTCGAAAATTTAGGAGGTTTATCG	Amplify *CD0183* for pRFP185 cloning
NF1799	GGGGGATCCTTATACTACTTTTCCTGCTTCAAATGTC	Amplify 2 x CwpV Type II repeats for cloning into pHAS037
NF1817	GGGGATCCTCCTCCCTCGAGTAATATTCTTTTTGCTGTAACAAATCTTGC	To amplify 0183 without its stop codon and introduce a XhoI site for pRPF185 cloning
NF1818	GGGCTCGAGTCATCAAAAGTAGATAAAGTAGTTTTGAAAAA	Amplify 2 x CwpV Type II repeats for cloning into pHAS037
NF2134	GATAGAGCTCAATGAAAGGAGCATTAGATTTATGAAAAA	Amplify *CD3392* without its stop codon for pHAS039 cloning
NF2135	GATACTCGAGTGATTTCTTCATTTTACGGCGTTTAT	Amplify *CD3392* without its stop codon for pHAS039 cloning
NF2288	GATAGGATCCTTATTTTGACTTTTTATCCTTTAACTCTTTTTG	Amplify *CD2537* for pRFP185 cloning
NF2378	GATAGAGCTCAAAATGAAAGGAGCATTAGATTTATG	Amplify *CD3392* for pRFP185 cloning
NF2379	GATAGGATCCTTATGATTTCTTCATTTTACGGC	Amplify *CD3392* for pRFP185 cloning
NF2581	TAAGATCCTATAAGTTTTAATAAAACTTTAAATAG	Remove sorting signal of *CD3392* by inverse PCR
NF2582	GTCGCTAGGTCTGCTAGG	Remove SPKTG sorting motif or sorting signal of *CD3392* by inverse PCR
NF2643	GATAGAGCTCCTAATTTGAGGAGGCTAATTTATATTG	Amplify *CD2537* for pRFP185 cloning
NF2722	TTCCAGATTATGCTGAGCGATTTGAAGAAATAACCATTTTT	Add a sequence encoding for HA tag into the *CD2537* by inverse PCR
NF2723	CATCATATGGATATAGCCCATAAGAAATAAAACTCTGAC	Add a sequence encoding for HA tag into the *CD2537* by inverse PCR
NF2855	TTTTTCTTTAGCTTCTCTTTTAGATATTCC	Remove SPKTG sorting motif or sorting signal of *HA-CD2537* by inverse PCR
NF2856	GATTTAGGTTTTTCAAATAGTATAATTATATTTATAG	Remove SPKTG sorting motif of *HA-CD2537* by inverse PCR
NF2857	TAAGGATCCACTAGTAACGGC	Remove sorting signal of *HA-*

		CD2537 by inverse PCR
NF2863	GATCCTCGAGTTTTGACTTTTT ATCCTTTAACTCTTTTTGATT	Amplify *HA-CD2537* without its stop codon for pHAS039 cloning
NF2883	GATC<u>GAGCTC</u>GAAAGGATTA GGTATTTGAGATTATG	Amplify *CD2768* for pRFP185 cloning
NF2884	GATC<u>GGATCC</u>TTATTGTAAAA CACGTGTAGCAGTTATAAG	Amplify *CD2768* for pRFP185 cloning
NF2887	TTCCAGATTATGCTACTAATT CACCTATGAGTGCAAC	Add a sequence encoding for HA tag into the *CD2768* by inverse PCR
NF2888	CATCATATGGATATGGCTCTG CTGCACTAGC	Add a sequence encoding for HA tag into the *CD2768* by inverse PCR

*Underlined bases indicate engineered restriction sites

REFERENCES

1. Fagan, R. P., and Fairweather, N. F. (2011) *Clostridium difficile* has two parallel and essential Sec secretion systems. *J Biol Chem* **286**, 27483-27493
