# Supplementary material for: Disparate subcellular location of putative sortase substrates in Clostridium difficile
Source: Sci Rep. 2017 Aug 23;7:9204. doi: 10.1038/s41598-017-08322-1 (PMC5569036; doi:10.1038/s41598-017-08322-1)
Supplement: Supplementary file 2 — 2 supplementary pdf [file 41598_2017_8322_MOESM2_ESM.pdf]

Disparate subcellular location of putative sortase substrates in *Clostridium difficile*

Johann Peltier, Helen A. Shaw, Brendan W. Wren and Neil F. Fairweather

Supplementary Information:

Supplementary Figures S1-S5: uncropped Western-blot images

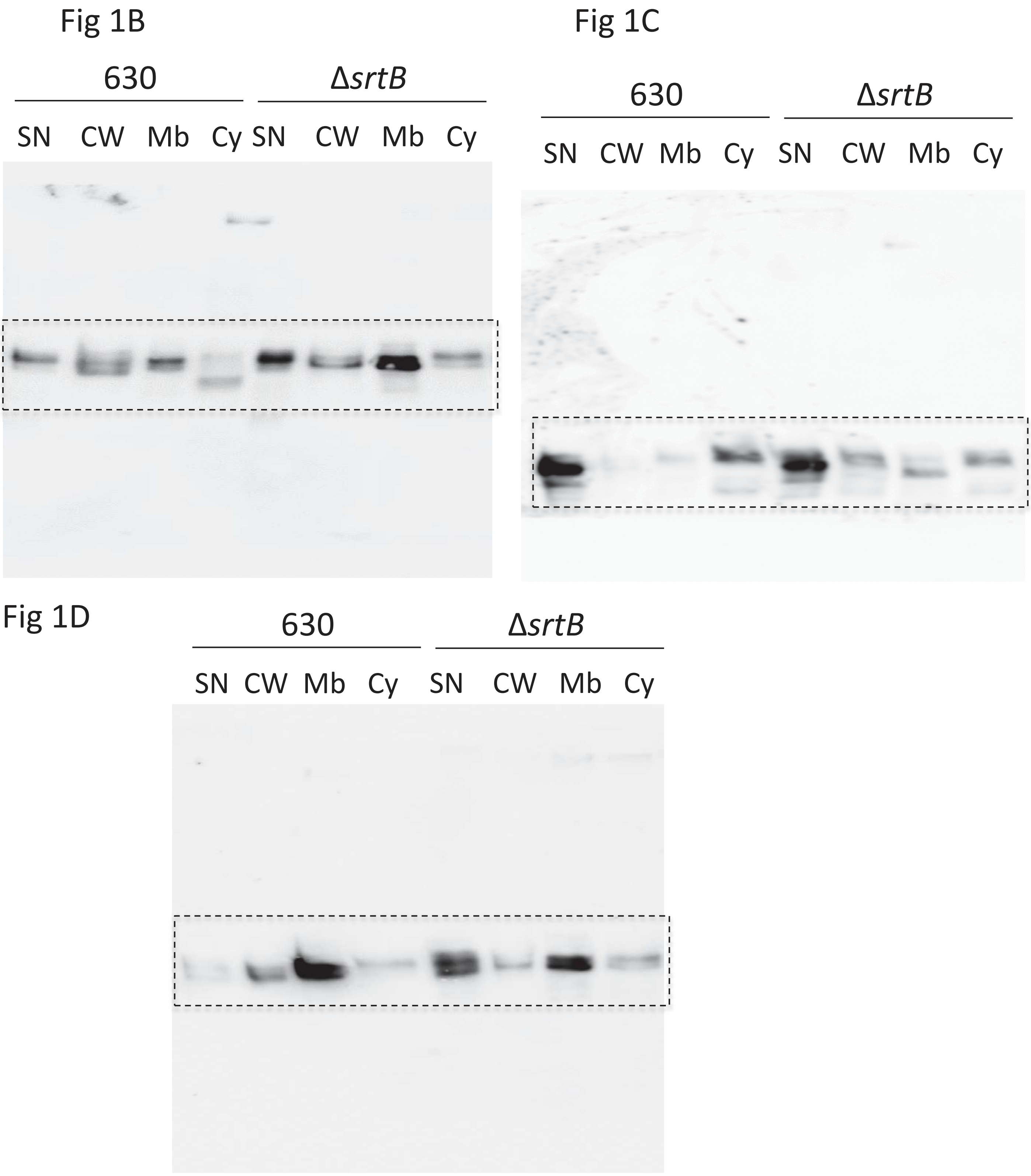

Supplementary Figure S1: Uncropped Western-blots images corresponding to Figure 1B, 1C and 1D.

Fig 2B

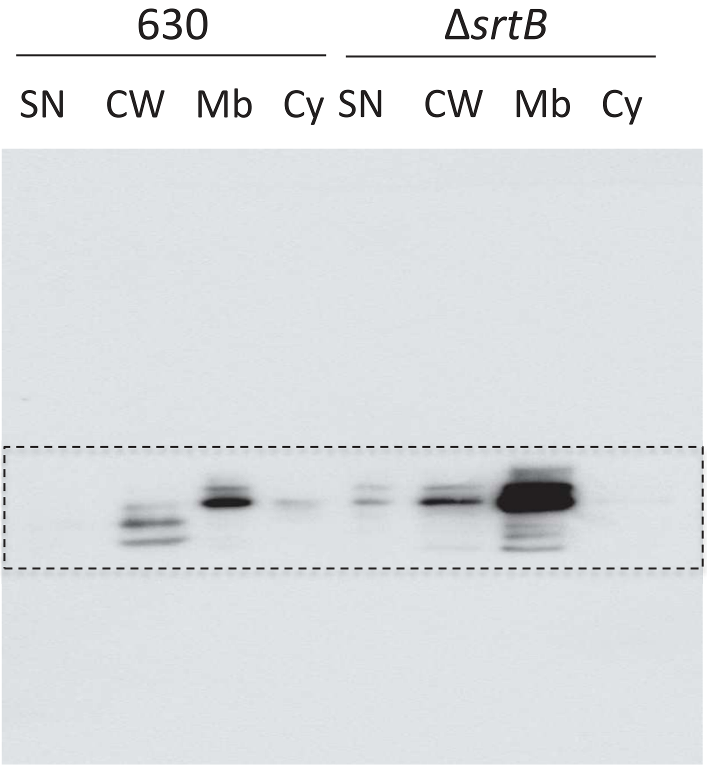

Fig 2C

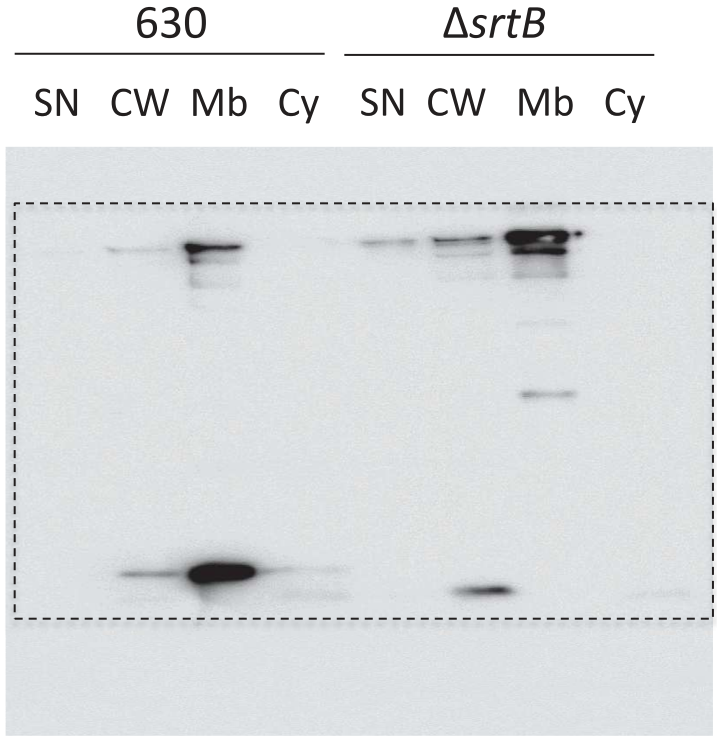

Supplementary Figure S2: Uncropped Western-blots images corresponding to Figure 2B and 2C.

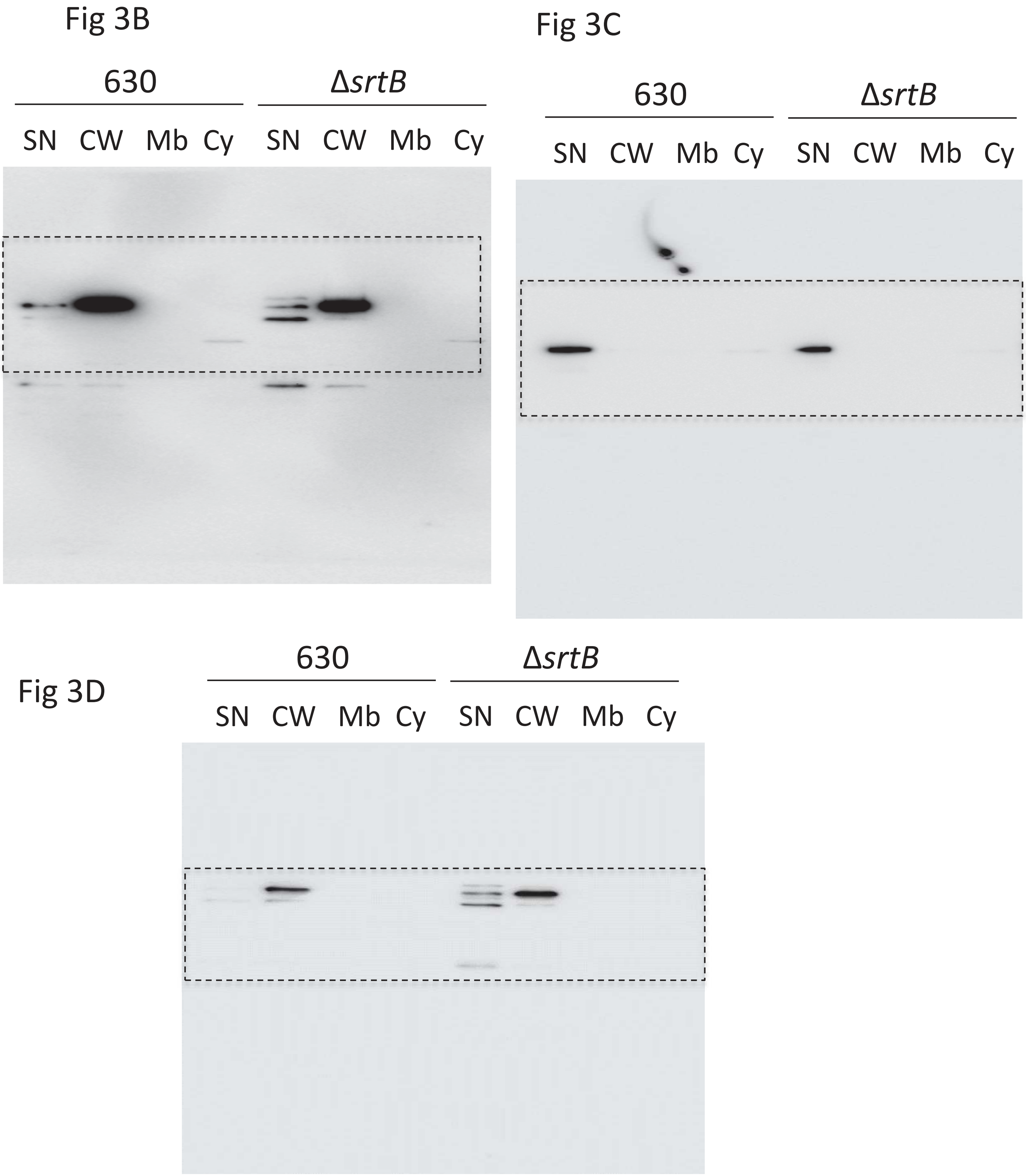

Supplementary Figure S3: Uncropped Western-blots images corresponding to Figure 3B and 3C and 3D.

Fig 4A

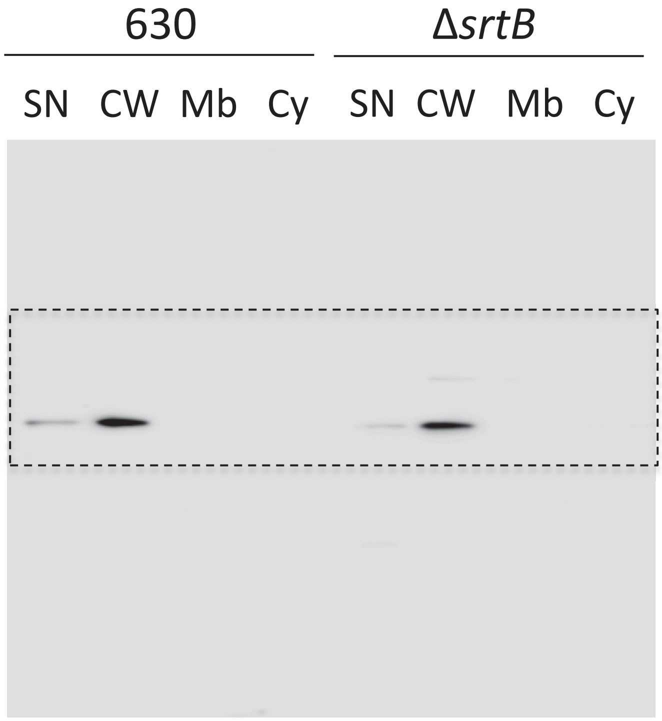

Fig 4B

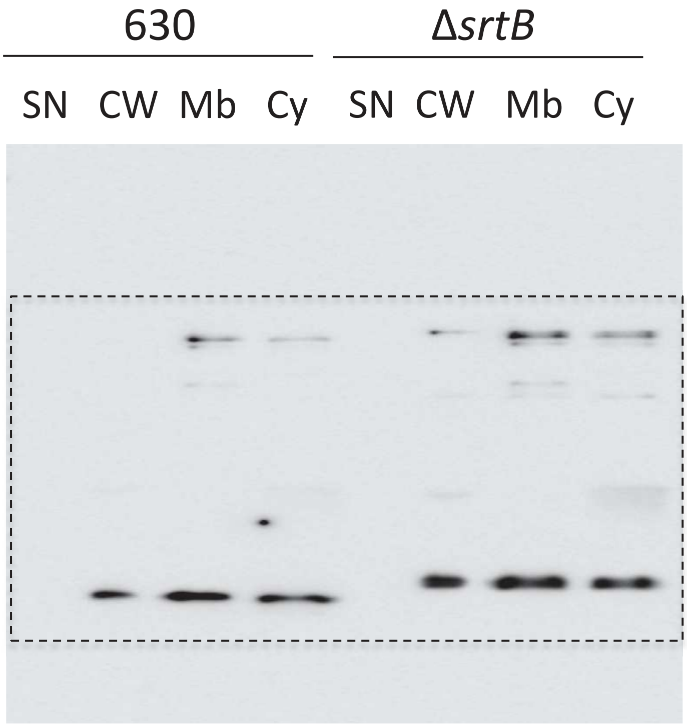

Supplementary Figure S4: Uncropped Western-blots images corresponding to Figure 4A and 4B.

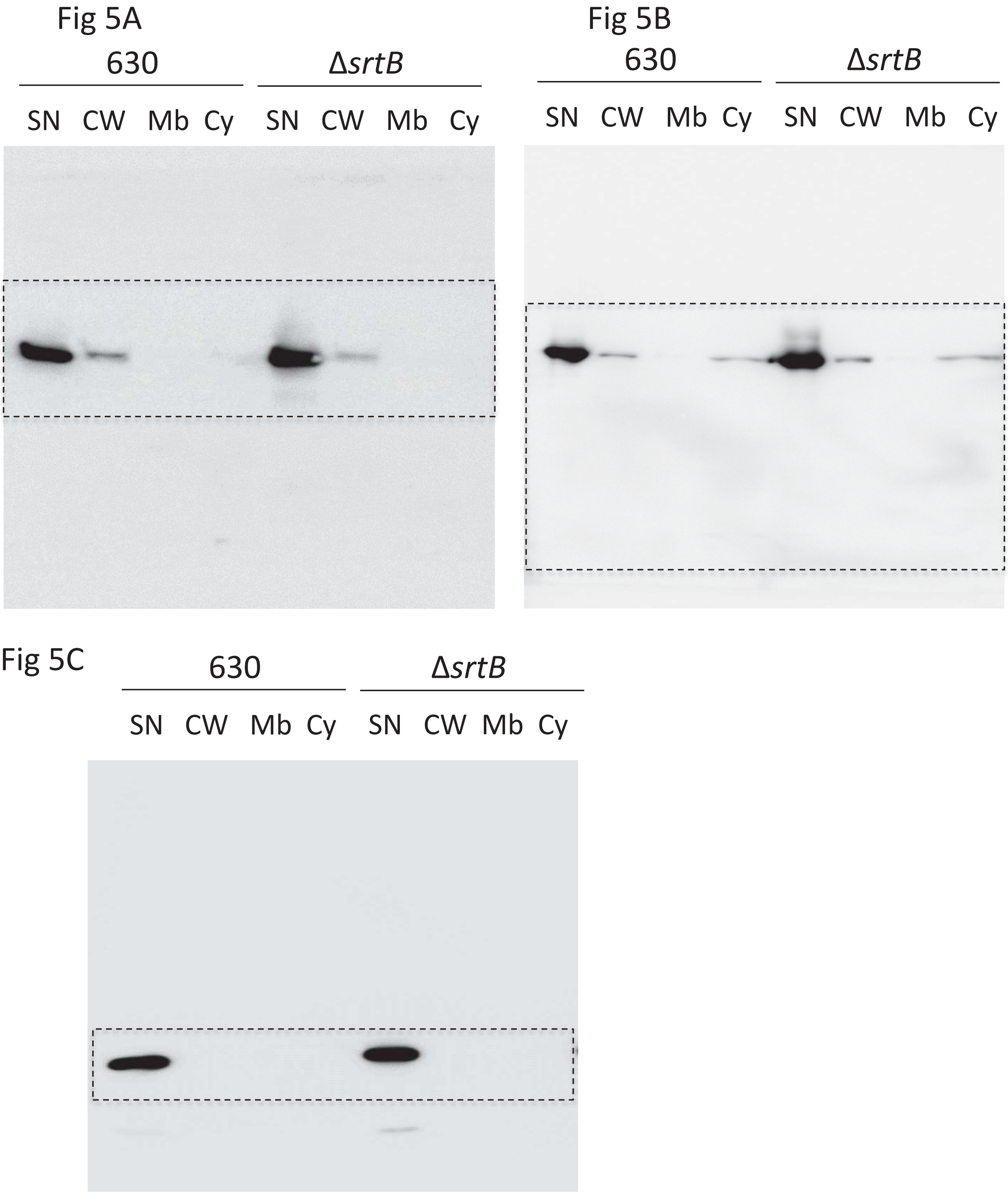

Supplementary Figure S5: Uncropped Western-blots images corresponding to Figure 5A, 5B and 5C.
